# Hodgkin’s lymphoma emerging radiation treatment techniques: trade-offs between late radio-induced toxicities and secondary malignant neoplasms

**DOI:** 10.1186/1748-717X-8-22

**Published:** 2013-01-30

**Authors:** Laura Cella, Manuel Conson, Maria Cristina Pressello, Silvia Molinelli, Uwe Schneider, Vittorio Donato, Roberto Orecchia, Marco Salvatore, Roberto Pacelli

**Affiliations:** 1 Institute of Biostructures and Bioimaging, National Council of Research (CNR), Napoli, Italy; 2 Department of Diagnostic Imaging and Radiation Oncology, University “Federico II” of Napoli, Napoli, Italy; 3 Department of Health Physics, S. Camillo-Forlanini Hospital, Roma, Italy; 4 Unit of Medical Physics, Centro Nazionale di Adroterapia Oncologica Foundation, Pavia, Italy; 5 Vetsussie Faculty, University of Zürich and Radiotherapy, Hirslanden, Aarau, Switzerland; 6 Department of Radiation Oncology, S. Camillo-Forlanini Hospital, Roma, Italy; 7 Advanced Radiotherapy Center, European Institute of Oncology, Milano, Italy

**Keywords:** Hodgkin’s lymphoma, Emerging radiotherapy techniques, Radio-induced toxicity, Second malignant neoplasm

## Abstract

**Background:**

Purpose of this study is to explore the trade-offs between radio-induced toxicities and second malignant neoplasm (SMN) induction risk of different emerging radiotherapy techniques for Hodgkin’s lymphoma (HL) through a comprehensive dosimetric analysis on a representative clinical model.

**Methods:**

Three different planning target volume (PTV_i_) scenarios of a female patient with supradiaphragmatic HL were used as models for the purpose of this study. Five treatment radiation techniques were simulated: an anterior-posterior parallel-opposed (AP-PA), a forward intensity modulated (FIMRT), an inverse intensity modulated (IMRT), a Tomotherapy (TOMO), a proton (PRO) technique. A radiation dose of 30 Gy or CGE was prescribed. Dose-volume histograms of PTVs and organs-at-risk (OARs) were calculated and related to available dose-volume constraints. SMN risk for breasts, thyroid, and lungs was estimated through the Organ Equivalent Dose model considering cell repopulation and inhomogeneous organ doses.

**Results:**

With similar level of PTV_i_ coverage, IMRT, TOMO and PRO plans generally reduced the OARs’ dose and accordingly the related radio-induced toxicities. However, only TOMO and PRO plans were compliant with all constraints in all scenarios. For the IMRT and TOMO plans an increased risk of development of breast, and lung SMN compared with AP-PA and FIMRT techniques was estimated. Only PRO plans seemed to reduce the risk of predicted SMN compared with AP-PA technique.

**Conclusions:**

Our model–based study supports the use of advanced RT techniques to successfully spare OARs and to reduce the risk of radio-induced toxicities in HL patients. However, the estimated increase of SMNs’ risk inherent to TOMO and IMRT techniques should be carefully considered in the evaluation of a risk-adapted therapeutic strategy.

## Background

In the past decades, treatment improvements have made Hodgkin’s lymphoma (HL) one of the most curable malignancies. However, due to the low patients mean age, the combined use of potentially harmful therapeutic agents and the efficiency of the therapy that allows a high cure rate with a long life span expectation, late effects of HL treatment represent an important and considerable threat for surviving patients. Indeed, the older series of successfully treated long term surviving patients showed a high rate of late side effects of therapy including iatrogenic lung, heart and thyroid diseases [1-3].

Technological advances in HL radiation therapy (RT) [4-11] by high conformal treatments potentially increase control over organs-at-risk (OAR) dose distribution. Dose-volume histogram (DVH) predictors in HL patients have been reported for late side effects such as radiation pneumonitis [12], hypothyroidism [13], and cardiovascular diseases [14,15] supporting the planning optimization procedures so as to limit OAR complication risks.

However, considering the low mean age, the high cure rate, and the consequent long survival expectation of HL patients, caution must be taken in the application of modern techniques such as intensity modulated radiotherapy or Tomotherapy because of the greater volume of normal tissue receiving low-to-moderate radiation doses and their inherent risk of second malignant neoplasms (SMNs) that may be significantly higher compared with 3D conformal radiotherapy [16]. Moreover, the impact on SMN incidence from particle therapy producing secondary neutrons causes some concern [17]. Structures with a high potential for the development of second malignancies, such as lung, thyroid and breast, must be considered.

Predicting SMN risk from these newer and sophisticated RT delivery techniques is complicated by their having been only recently introduced and by the consequent absence of epidemiological data [18]. As an alternative, biologically-based mathematical models can be used to estimate the risk of SMNs related to a given RT technique using organ dose distribution through dose-volume histograms [19-23]. These models allow to compare dose distributions with regard to the estimated risk of SMNs in the irradiated organs as a function of point dose in the radiotherapy dose range also including fractionation effects.

The aim of this study is to analyze normal tissue sparing capability of different RT techniques for one representative supradiaphragmatic HL model case, in particular to explore the trade-offs between radio-induced toxicities and SMNs induction risk. For this purpose, we have conceived three different size planning target volumes (PTVs), each with different involvement of OARs such as heart, thyroid, breasts and lungs. We have simulated RT plans using five different delivery techniques. DVHs were then used to predict the impact of the different analysed RT techniques on late side effects and on SMN induction risk estimated through the Organ Equivalent Dose (OED) model considering cell repopulation and inhomogeneous organ doses [20].

## Methods

**Figure 1:**
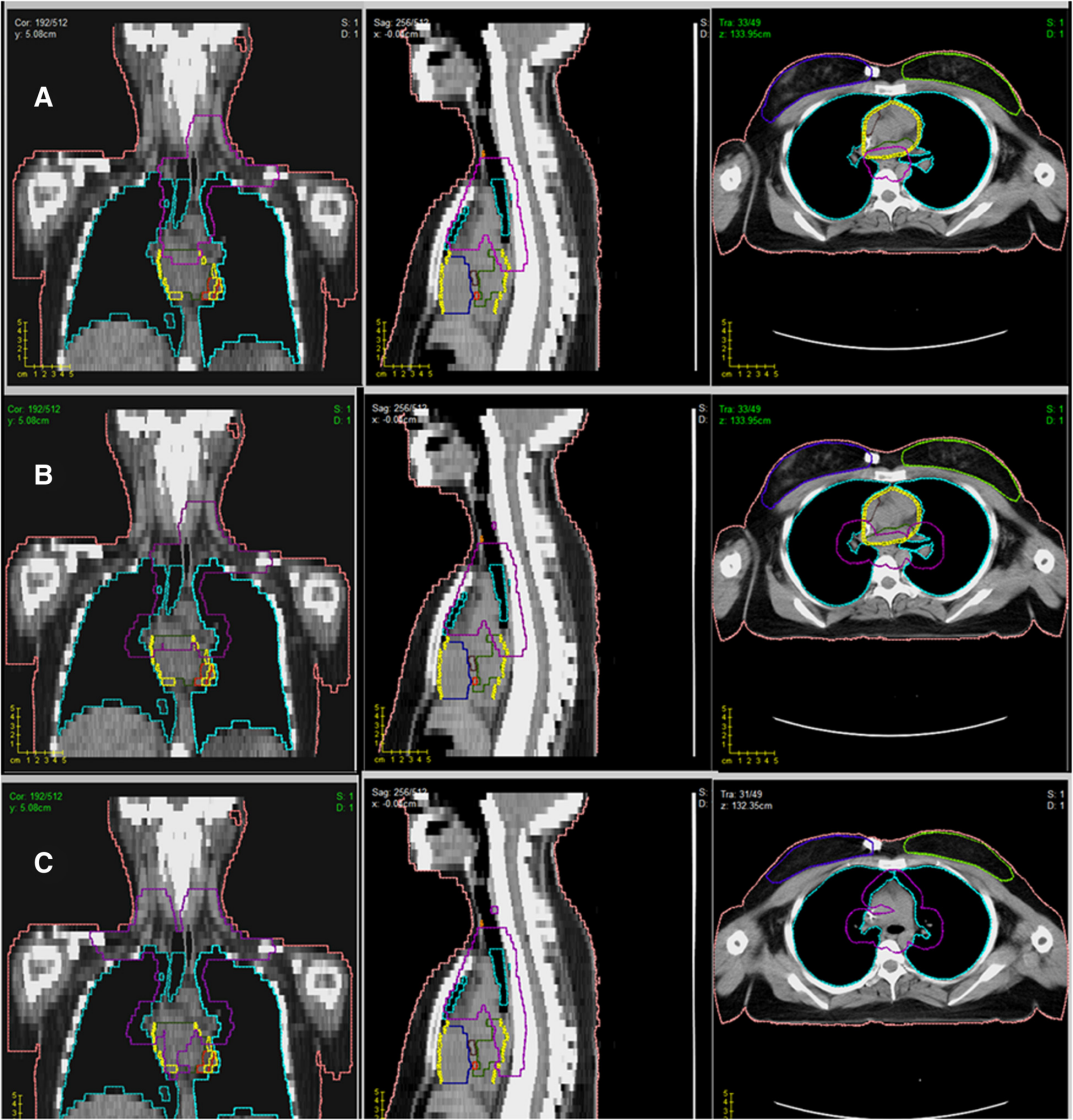
**Three different planning target volume size scenarios: A) small (PTV**_**1**_**), B) medium (PTV**_**2**_**), C) large (PTV**_**3**_**).** PTVi are outlined in purple.

Planning CT-scan of a female patient with supradiaphragmatic HL in standard supine position with 5-mm slices acquisition was considered. Different involved field clinical target volume (CTV_i_) size scenarios were generated: small (CTV_1_), medium (CTV_2_), and large (CTV_3_). The CTV_1_ included the upper mediastinal and left supraclavear nodal sites; the CTV_2_ included CTV_1_ plus bilateral lung hylus; the CTV_3_ included the whole mediastinum, the bilateral lung hylus, and bilateral supraclavear nodal sites. The nodal sites were delineated as described elsewhere [24]. Planning target volumes (PTV_i_) included CTV_i_ plus a 10 mm margin (Figure 1). The following OARs were contoured: bilateral lungs, whole heart, cardiac chambers, pericardium, thyroid and breasts. For cardiac structures delineation, the heart atlas [25] was applied while breasts were defined as described by Weber *et al.*[26].

### Radiotherapy techniques

Five treatment plans were generated on purpose for each PTV_i_: a conventional anterior-posterior parallel-opposed (AP-PA) plan, a forward intensity modulated plan (FIMRT), an inverse intensity modulated plan (IMRT), a Tomotherapy plan (TOMO), and a proton plan (PRO). A total dose of 30 Gy or cobalt gray equivalent (CGE) in 20 daily fractions of 1.5 Gy was planned. All treatment plans were optimized to ensure 95% of the prescription dose delivered at least to 95% of the PTV with a maximum dose less than 115%.

#### AP-PA

Conventional AP-PA plans were simulated using photon beams from a linac equipped with 40 pairs of multileaf collimator (MLC). Treatment planning was performed by a 3-D planning system (XiO, Elekta-CMS) and a convolution dose calculation algorithm was applied.

#### FIMRT

A step-by-step iterative process inherent to forward planning was used manually adding two or more MLC shaped subfields with the same AP-PA isocenter and gantry position. Treatment plans were generated with XIO planning system; the MLC positions and beam weightings were optimized by forward planning based on the 3D dose distribution as well as on DVHs.

#### IMRT

Seven field IMRT treatments were planned with Pinnacle3 TPS (Philips) using Direct Machine Parameter Optimization and Cone Convolution algorithm (CCA) for dose calculation and a Siemens Artiste linac, with a step-and-shoot technique performed with the 160 leaves collimator.

#### TOMO

Tomotherapy treatments were planned with Tomotherapy Planning Station (Accuray) with gradient descent optimization algorithm, establishing a 2.5 field width, 0,287 pitch value and a starting Modulation Factor (MF) of 4 and an actual MF of 3.9, 3.6 and 2.7 for PTV_1_, PTV_2_, and PTV_3_ plans, respectively. The dose distributions were calculated with CCA. Delivery was performed with fifty-one fields for each gantry rotation and beam modulation carried out with a 64 leaves binary collimator.

#### PRO

Proton plans were generated with the Syngo RT Planning Station (Siemens VB-10), using an active scanning dose delivery system (Centro Nazionale di Adroterapia Oncologica Foundation). Proton energies from 62 to 180 MeV/u were used with a nominal 10 mm Full Width Half Maximum pencil beam focus and a beam intensity of 2*10^9^ particles per spill. A scanning step of 3 mm was fixed for the transversal directions and a 2 mm energy step was selected for Spread Out Bragg Peak generation. For PTV_1_ and PTV_2_, an AP-PA configuration was defined, while two couples of parallel-opposed beams, centered on the PTV, were applied for PTV_3_. A 4 cm range shifter was introduced for the anterior beam directions to achieve the minimum proton energy required. A fixed RBE value of 1.1 was used.

IMRT, TOMO and PRO plans were optimized using constraints on the OARs and priority weightings published by Weber et al. [26].

All treatment characteristics including the delivered monitor units and the number of used protons are summarized in Table 1. The contribution from the scattered neutrons was considered and the dose distribution corrected using the data from d’Errico *et al.*[27] for photons, and the data from Schneider et al. [28] for spot-scanned protons, for neutron equivalent dose estimation. The following neutron equivalent dose in Sv per applied MUs and per treatment protons per Gy were used: H^*N,6MV*^= 0, H^*N,15MV*^= 1x10^-5^, H^*N,protons*^= 6x10^-14^. The out-of-axis neutron dose contribution was neglected since the OARs we considered for plan evaluation were included in the primary dose distribution. However, for organs far from the target volume which were not considered in the present study the out-of-field neutron contribution can be important.

**Table 1:** Treatment techniques characteristics

***Technique- PTV***_***i***_	***Fields or Subfields number***	***Energy (MeV)***	***Total MUs or protons per Gy***	***Neutron equivalent dose (Sv)***
AP-PA- PTV_1_	2	6	5040	0
AP-PA- PTV_2_	2	6	5038	0
AP-PA- PTV_3_	2	6	4157	0
FIMRT- PTV_1_	1	6	1427	0
	3	15	2070	0.020
FIMRT- PTV_2_	1	6	1415	0
	3	15	2040	0.020
FIMRT-PTV_3_	3	6	1714	0
	3	15	2277	0.020
IMRT- PTV_1_	7	6	8020	0
IMRT- PTV_2_	7	6	9500	0
IMRT- PTV_3_	7	6	14000	0
TOMO- PTV_1_		6	10042	0
TOMO- PTV_2_		6	9661	0
TOMO- PTV_3_		6	8232	0
PROTONS- PTV_1_	1	88-170	5.43*10^10^	0.16
	1	62-162	4.52*10^10^	
PROTONS- PTV_2_	1	87-171	6.20*10^10^	0.19
	1	62-162	4.52*10^10^	
PROTONS- PTV_3_	1	88-180	3.52*10^10^	0.23
	1	88–173	3.92*10^10^	
	1	62–162	3.42*10^10^	
	1	62-166	3.34*10^10^	

### Plan evaluation

For each RT technique and for each PTV scenario specific organ dose-volume metrics and dose parameters were calculated from DVHs and related to available predictors for radio-induced toxicities:

Whole Heart: V25 <10% [15]; endpoint: long-term cardiac mortality;

Cardiac Chambers: Left Atrium V25<63%; Left Ventricle V30<25%; Right Ventricle V30≤65% [14]; endpoint: asymptomatic heart valvular dysfunction;

Pericardium: V30≤46%; mean dose ≤26 Gy [15]; endpoint: pericarditis;

Lungs: V20≤33.5%; mean dose ≤13.5 Gy [12]; endpoint: symptomatic radiation pneumonitis;

Thyroid: V30≤62% [13]; endpoint: clinical or subclinical hypothyroidism.

Where VX is the percentage of organ volume exceeding X Gy.

Of note, the threshold metrics for cardiac chambers, lungs, and thyroid were specifically extrapolated from HL patients’ cohorts.

The DVHs were also utilized to estimate the SMN risk for breasts, lungs and thyroid through the application of the OED concept using specific organ model-parameters. Accordingly, the risk ratio (RR) for a RT plan *i* relative to another plan *j* with respect to cancer induction in one organ is equivalent to the OED ratio [20]:

(1) $${\mathrm{RR}}_{\mathrm{ij}}=\frac{\mathrm{OED}\;\left(\mathrm{pla}n_{i}\right)}{\mathrm{OED}\;\left(\mathrm{pla}n_{j}\right)}$$

The AP-PA plan was used as reference for RR_ij_ calculation.

The OED can be determined on the basis of an organ specific dose–response relationship for radiation induced cancer (“risk equivalent dose”, RED) and DVH. The RED for carcinoma induction is given by a mechanistic model accounting for cell killing and fractionation effects [19]. Briefly, for a given organ and a given plan:

(2) $$\mathrm{OED}=\frac{1}{V_{T}}\underset{i}{\sum }V\left(D_{i}\right)\mathrm{RED}\left(D_{i}\right)$$

where V_T_ is the total organ volume and the sum is taken over all DVH bins, and

(3) $$\mathrm{RED}\left(D\right)=\frac{e^{-\alpha^{\prime }D}}{\alpha^{\prime }R}\left(1-2R+R^{2}e^{\alpha^{\prime }D}-{\left(1-R\right)}^{2}e^{-\frac{\alpha^{\prime }R}{1-R}D}\right)$$

where *α’=α+βd*, with *α* and *β* denoting the linear-quadratic model parameters for the organ of interest and *d* the dose fraction, *D* the total dose, and *R* the repopulation/repair parameter. The dose–response model is robust with variations in *α/β*[20] and an *α/β*= 3 Gy was used for all calculations. The risk for secondary breast, thyroid and lung cancers were estimated with parameter values *α*= 0.067; 0.0318; 0.042 Gy^-1^ and *R*= 0.62; 0.0; 0.83 respectively [20]. Using *RED (D)* given by eq. (3), the risk-volume histograms (RVHs) for breast, thyroid and lung cancers were calculated.

## Results

Target sizes were PTV_1_= 497.0 cm^3^, PTV_2_ = 626.7 cm^3^, and PTV_3_ = 837.4 cm^3^. All RT techniques succeeded in obtaining the requested PTV_i_ dose coverage independently of PTV size. Comparative DVHs for the different PTV_i_ and for all techniques are shown in Additional file 1. PTV_i_ coverage was optimal with both TOMO and PRO plans.

### Radiation dose to OARs

In Table 2 are reported the DVH parameters for the different RT plans and for each PTV_i_. With regard to PTV_1_ and PTV_2_ scenarios, DVH analysis (Additional file 2a and 2b) shows that all the different techniques respected the considered constraints with the exception of the whole-heart V25 for which only the TOMO and PRO plans were able to reduce it under 10%. Regarding the PTV_3_ the AP-PA, FIMRT, IMRT plans violate the dose-volume limits for the whole-heart and for the left atrium. In addition, the AP-PA plan exceeds the 62% volume for thyroid V30 and the IMRT plan shows a mean lung dose just equal to 13.5 Gy limit (Additional file 2c).

**Table 2:** **Organ dose-volume metrics and dose parameters for the different RT plans and for each PTV**_**i**_

		***AP-PA***	***FIMRT***	***IMRT***	***TOMO***	***PRO***
**PTV**_**1**_						
Heart	V25	22.5*	21.0*	11.8*	4.5	3.6
Left Atrium	V25	42.2	39.2	35.0	25.8	24.5
Left ventricle	V30	0.1	0.1	0.1	0.1	0.1
Right Ventricle	V30	7.6	2.4	0.1	0.1	0.1
Pericardium	V30	6.2	2.5	3.9	3.0	2.1
	mean dose	10.0	9.2	6.4	7.7	4.3
Thyroid	V30	49.0	48	41.4	16.9	28.5
Lungs	V20	12.1	11	15	10.1	8.2
	mean dose	5.3	4.9	7.8	8.1	3.3
**PTV**_**2**_						
Heart	V25	27.7*	29.9*	12.3*	5.0	6.7
Left Atrium	V25	41.8	45.0	41.5	27.2	30.4
Left ventricle	V30	9.2	7.5	0	0.1	0.3
Right Ventricle	V30	9.7	6.5	0	0	0
Pericardium	V30	14.9	11.5	9.2	5.5	2.9
	mean dose	11.8	12.5	10.7	7.8	7.5
Thyroid	V30	49.3	42.1	27.8	16	18.6
Lungs	V20	24.7	23.6	20.5	16.5	14.4
	mean dose	9.6	9.2	10.5	9.7	6.4
**PTV**_**3**_						
Heart	V25	60.5*	67.5*	22.0*	8.7	7.3
Left Atrium	V25	98.3*	99.3*	73.0*	49.4	43.0
Left ventricle	V30	12.0	13.0	2.5	0.1	0
Right Ventricle	V30	49.2	47.0	0	0	0
Pericardium	V30	31.3	42.0	20.6	13.0	3.2
	mean dose	22.0	23.8	17.2	14.6	10.2
Thyroid	V30	93.9*	24.0	60	45.0	7.0
Lungs	V20	30.7	28.8	27.0	23.0	14.5
	mean dose	11.9	11.1	13.5*	12.6	6.5

* not compliant with constraint.

VX is the percentage of organ volume exceeding X Gy.

In general, the PRO and TOMO plans provided the lowest parameter values for all the considered OARs and spared them better than IMRT plan.

### SMNs relative risk

**Figure 2:**
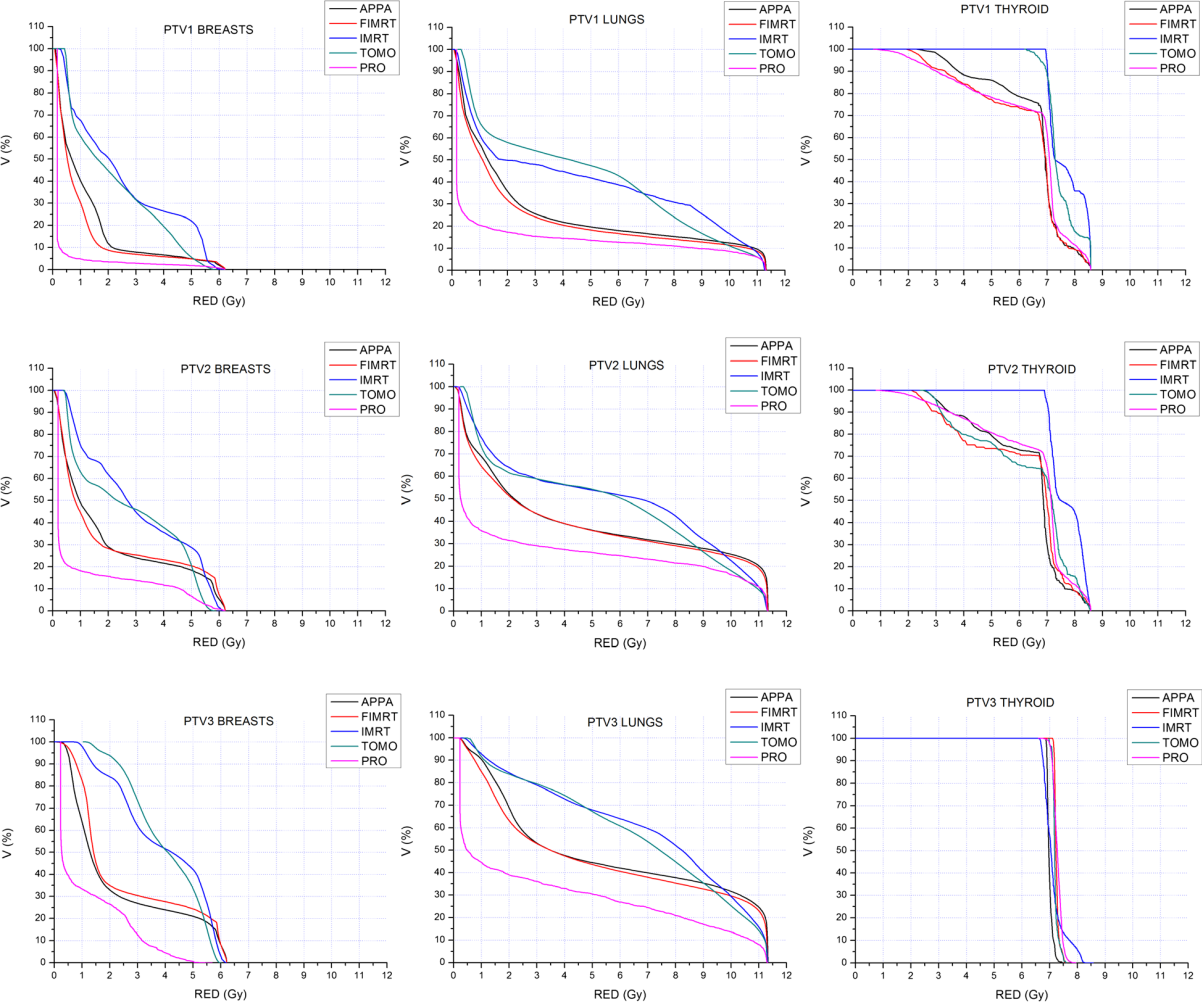
**Comparative risk-volume histograms for breasts, lungs and thyroid for each PTV**_**i**_**.**

The estimated OED values for breasts, lungs and thyroid for all RT techniques are listed in Table 3. In Figure 2 comparative RVHs for the above OARs are shown. In breasts and lungs, the PRO plan provided the lowest risk-volume curve whereas the highest curves were provided by IMRT and TOMO plans.

**Table 3:** **Estimated Organ Equivalent Dose (OED) for the different RT plans and for each PTV**_**i**_

	***PTV***_***1***_	***PTV***_***2***_	***PTV***_***3***_
	**OED**	**OED**	**OED**
**Breasts**			
AP-PA	1.09	1.41	2.25
FIMRT	0.94	1.92	2.52
IMRT	2.44	3.00	3.91
TOMO	2.13	2.71	4.02
PRO	0,35	0,94	1,14
**Lungs**			
AP-PA	2.82	4.48	5.58
FIMRT	2.64	4.38	5.34
IMRT	3.77	4.98	6.25
TOMO	4.60	5.35	6.76
PRO	1.67	2.98	3.30
**Thyroid**			
AP-PA	6.35	6.31	6.98
FIMRT	6.26	6.17	7.25
IMRT	7.69	7.71	7.12
TOMO	7.49	6.30	7.20
PRO	7.35	6.50	7.34

**Figure 3:**
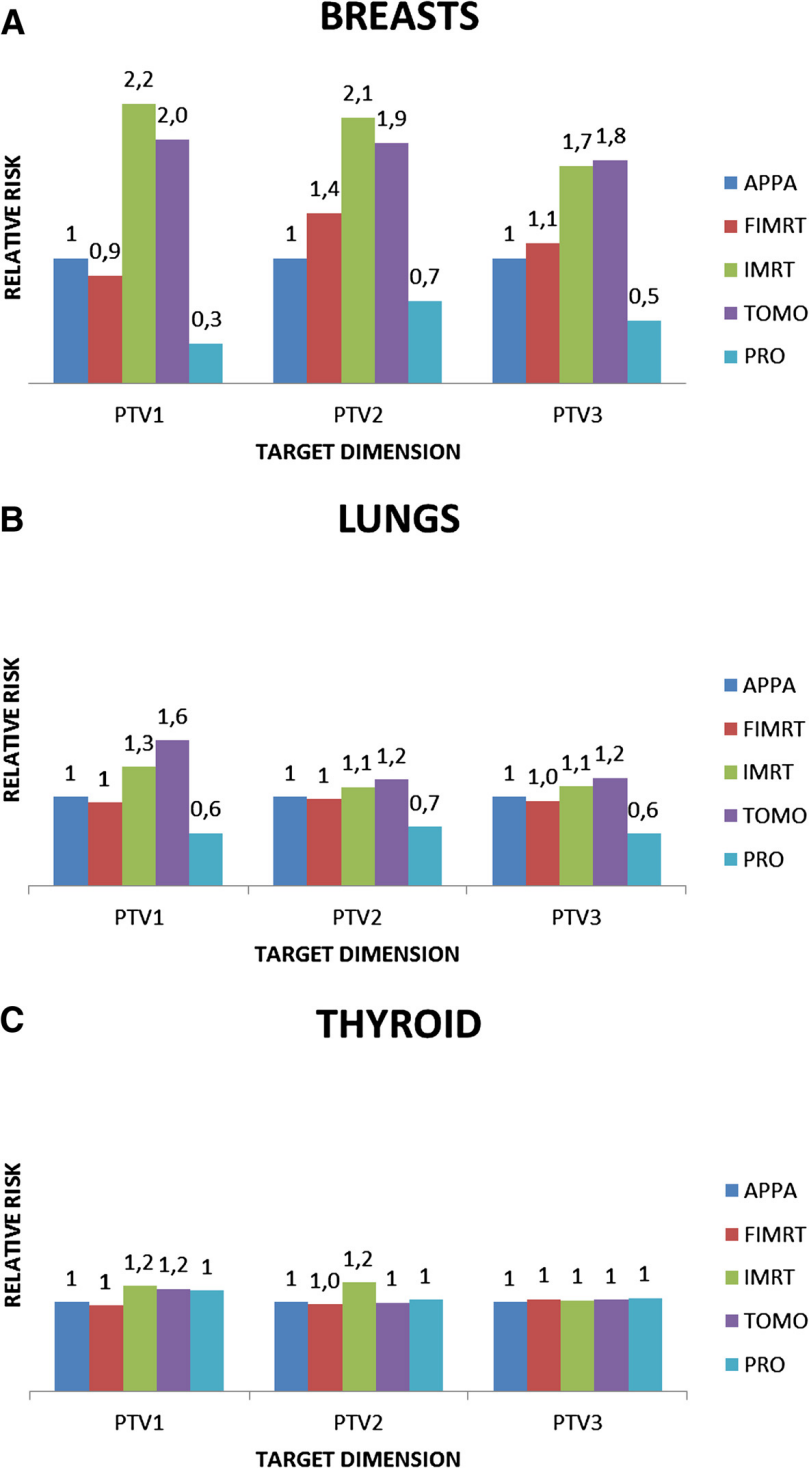
Estimated values of the risk ratio (RR) for FIMRT, IMRT, TOMO and PRO plans relative to the AP-PA plan with respect to SMN induction in breasts, lungs and thyroid.

RR values for FIMRT, IMRT, TOMO and PRO plans relative to the AP-PA plan with respect to SMN induction in breasts, lungs and thyroid are plotted in Figure 3.

Regarding breast cancer induction, in the PTV_1_ case, the IMRT and TOMO plans exhibit OED values of 2.44 and 2.13, respectively, which result in a RR for SMN induction that is 2.2- and 2.0-fold the risk of a AP-PA plan (Figure 3A). The IMRT and TOMO plans’ RR for breast cancer induction exhibit a small reduction when the PTV size increases; nonetheless we observe an approximately 2-fold increase compared with AP-PA or FIMRT techniques. On the contrary, in all PTV scenarios, PRO plan gives an OED in the range of 0.35-1.14 and accordingly a RR induction compared with conventional plan in the range of 0.3-0.7. When IMRT and TOMO were compared to PRO plan, we observed an increase in RR values for the breast by a factor ranging from a minimum of 3 (TOMO in PTV_2_) to a maximum of 7 (IMRT in PTV_1_).

For lung cancer induction (Figure 3B), we observed the same behavior for the TOMO and IMRT techniques increasing RR values by a factor 1.1-1.6 compared with the conventional plan, while with PRO plans we observed a RR reduction. Conversely, for thyroid (Figure 3C) the RR values are close to 1 for all the techniques and all target sizes except for IMRT in PTV_1_ and PTV_2_ cases and for TOMO in the PTV_1_ case in which the RR is 1.2.

## Discussion

Since the implementation in HL therapy of extended field irradiation, the high cure rate was offset by late side effects and development of SMNs in a relevant fraction of patients [1]. The progresses in imaging and the better knowledge of the disease biology, with consequent better prognostic stratification of patients, have allowed a decrease in the therapeutic load consisting in a progressive reduction of chemotherapy cycles, radiation dose and treated volume in most patients [29]. However, the risk of late iatrogenic effects remains remarkable. The radiation delivery techniques can heavily condition the distribution of the dose in tissues and alter the toxicity profile of a treatment. Involved-field IMRT has shown excellent target coverage and amelioration of side effects in a clinical study by Lu et al. [7]. Volumetric modulated arc therapy has been shown to significantly reduce hearth dose in HL patients affected with cardiovascular disease [30] and to perform better than IMRT in sparing the OARs when using involved nodal RT [10,26]. With the same purpose Tomotherapy has been recently proposed for the treatment of HL [8]. Recent preliminary studies of proton beam therapy for mediastinal HL have been reported [4]. The OARs toxicities and development of second breast neoplasms would be expected to be reduced by the use of particle therapy. However, while it is epidemiologically reasonable to expect that a dose reduction is associated with a reduced risk of late effects, an improvement in SMN risk due to dose reduction is not yet clearly established.

Our study aims at analyzing 5 different radiation delivery techniques in three different hypothetical scenarios of supradiaphragmatic HL through a comprehensive dosimetric study. The main endpoint was to investigate, for each single technique, the balance between the predicted OARs injuries and the predicted development of SMNs, with the same target optimal dose coverage. The advantage of IMRT for heart and left ventricle sparing as well as its disadvantages in the low dose region, in particular for breasts, have been already reported in the literature [9]. However, in our study the above advantages and disadvantages were quantified and extended to other state-of-the-art techniques.

As surrogate indicators of OARs morbidities, some of the constraints recently suggested by the literature were used. We chose constraints predictive of feared radioinduced injuries commonly described in patients treated with sequential chemo-radiotherapy for HL such as hypothyroidism [13], asymptomatic cardiac valvular dysfunction [14] and radiation pneumonitis [12] and specifically extrapolated from HL patients’ cohorts, together with some other more general constraints suggested by QUANTEC reviews [15].

Lacking epidemiological data relative to the recent RT delivery techniques, estimation of the risk of SMN for breasts, lungs, and thyroid based on mathematical models [19,20] was used. Many uncertainties are involved in modelling the underlying biology of radiation induced-cancer. Nevertheless, these models may be reliably used to predict the impact on SMN induction of a given technique relative to another reference technique. To this end, we introduced the concept of risk ratio RR as a parameter for plan evaluation.

It should be noted that for the quality factor *Q* (stochastic RBE) we take a value of 1.1 for protons. There is a strong energy dependence for the quality factor and a factor of two, as recommended by ICRP 92 [31], would perhaps only be expected at very low energies in the tail of the Bragg peak. The main contribution to the normal tissue integral dose, however, will come from the plateau region of the Bragg curve due to the protons passing through normal tissue to reach the target volume. This portion of the Bragg curve consists predominantly of dose deposited by higher energy protons (much higher than 8 MeV) for which the NCRP quotes a value of one [32,33]. In the normal tissue distal to the target volume, although the quality factor may be higher, the irradiated volume will be very much smaller and the deposited dose will be lower due to the finite maximum range of protons in the tissue. Therefore, it is safe to assume that the vast majority of normal tissues will be irradiated by protons with a quality factor close to one.

As regards PTV coverage, in the framework of a satisfactory performance of all the above techniques, the optimal coverage was obtained by TOMO and PRO plans.

As far as constraint compliance is concerned, in all PTV scenarios, AP-PA, FIMRT, and IMRT plans exceed the whole-heart-V25 of 10%. This limit, associated with a <1% probability of cardiac mortality, is an overly safe risk estimate based on model predictions and consequently the risk may be overestimated [15]. The other constraints were met by all five techniques in PTV_1_ and PTV_2_ scenarios. For PTV_3_, the AP-PA, FIMRT and IMRT also failed to meet left atrium V25 cutoff volume of 63% which is a significant predictor of mitral and aortic valvular defects. The latter are particularly important for those patients characterized by high cure rates and prolonged survival like HL patients because of their progressive nature and potential contribution to overt cardiac toxicity [14]. Only the AP-PA failed to meet thyroid V30 dose constraint predictive of hypothyroidism. Remarkably, beyond DVH predictors, TOMO and PRO led to a reduction in the doses to all the OARs compared with the other plans.

Conversely, the estimated risk ratio of SMNs induction for breasts and lungs was significantly increased by IMRT and TOMO in all scenarios though it is lower when the target volume is larger. No relevant risk ratio increase in thyroid cancer was found for any technique. To be noted, theoretically PRO led to a reduction of risk ratio in all cases. Among photon delivery techniques, conventional AP-PA and FIMRT resulted in the lowest estimated risk of SMNs.

This study, exploring the trade-offs between radio-induced toxicities and SMN by planning comparative evaluations, provides informative tools so as to evaluate which HL patient potentially deserves a more advanced radiation technique obtaining a real advantage in terms of deterministic and/or stochastic damage prevention. Diverse variables must be considered such as individual patients features, site and size of disease in order to establish strategies capable of performing a risk-adapted radiotherapy.

Let us point to some potential limitations of our proof-of-concept study. First, we considered one single model case not taking into account morphological differences peculiar to each single patient such as heart, lung and breast volumes. We also analyzed three different PTVs that, although paradigmatic, did not cover all possible varieties of HL. Moreover, in SMN estimation the uncertainty linked to neutron RBE for carcinogenesis should be taken into account .

Given the above considerations, our analysis suggests that, as already shown for other tumor sites [17,34], proton therapy could theoretically be the optimal radiation modality in all HL scenarios studied, provided that plan robustness and organ motion are properly managed [35]. However, costs and availability currently limit proton usage. Regarding photon techniques, the choice of the more appropriate treatment should be tailored to the individual case. For instance, for a young male patient with a large tumor or a patient with cardiac co-morbidity both requiring a total dose of 30 Gy, TOMO plan would result extremely advantageous. On the contrary, TOMO could not be equally advantageous for a good prognosis young (25 years) HL bearing female patient requiring a total dose of 20 Gy, which implies a very low risk of late organ injuries. In such a case, radioinduced breast cancer may be of more concern and FIMRT may result more appropriate.

## Conclusions

Our model–based study fosters the use of advanced RT techniques to reduce the dose to OARs and, consequently, the risk of radio-induced toxicities in HL patients. However, in the framework of a modern risk-adapted therapeutic strategy, the estimated increase of SMNs’ risk inherent to TOMO and IMRT techniques should be carefully considered.

## Abbreviations

AP-PA: Anterior-posterior parallel opposed; CCA: Cone convolution algorithm; CGE: Cobalt Gray equivalent; CTV: Clinical target volume; DVH: Dose-volume histogram; FIMRT: Forward intensity modulated radiation therapy; FWHM: Full width at half maximum; IMRT: Inverse intensity modulated radiation therapy; HL: Hodgkin’s lymphoma; MF: Modulation factor; MLC: Multi-leaf collimator; OAR: Organ-at-risk; OED: Organ equivalent dose; PRO: Proton; PTV: Planning target volume; RED: Risk equivalent dose; RR: Risk ratio; RT: Radiation therapy; RVH: Risk-volume histograms; SMN: Second malignant neoplasm; TOMO: Tomotherapy

## Competing interests

The authors declare no conflict of interest.

## Authors’ contributions

LC and RP conceived and designed the study. MC, MS and RP defined target volumes. LC, MC and RP performed conventional technique plans. MCP and VD performed IMRT and TOMO plans. SM and RO performed proton plans. LC and US applied second cancer modeling. LC reviewed and analyzed all dosimetric data. All authors participated in drafting and revising the manuscript. All authors have given their final approval of the manuscript.

## Supplementary Material

Additional file 1 **Comparative dose-volume histograms for each PTV**_i_**scenario and for all techniques.** Click here for file

Additional file 2 **Comparative dose-volume histograms for each organ-at-risk and for all techniques for a) PTV**_1_** scenario, b) PTV**_2_**scenario, c) PTV3 scenario.** Click here for file
