# Supplementary material for: Hodgkin’s lymphoma emerging radiation treatment techniques: trade-offs between late radio-induced toxicities and secondary malignant neoplasms
Source: Radiat Oncol. 2013 Jan 30;8:22. doi: 10.1186/1748-717X-8-22 (PMC3641014; doi:10.1186/1748-717X-8-22)
Supplement: Additional file 2 — Comparative dose-volume histograms for each organ-at-risk and for all techniques for a) PTV1 scenario, b) PTV2scenario, c) PTV3 scenario. [file 1748-717X-8-22-S2.docx]

a)

c)

b)
