# Supplementary material for: Hodgkin’s lymphoma emerging radiation treatment techniques: trade-offs between late radio-induced toxicities and secondary malignant neoplasms
Source: Radiat Oncol. 2013 Jan 30;8:22. doi: 10.1186/1748-717X-8-22 (PMC3641014; doi:10.1186/1748-717X-8-22)

PTV coverage

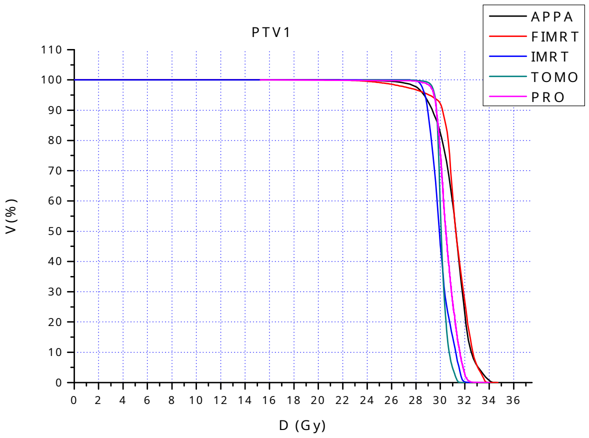

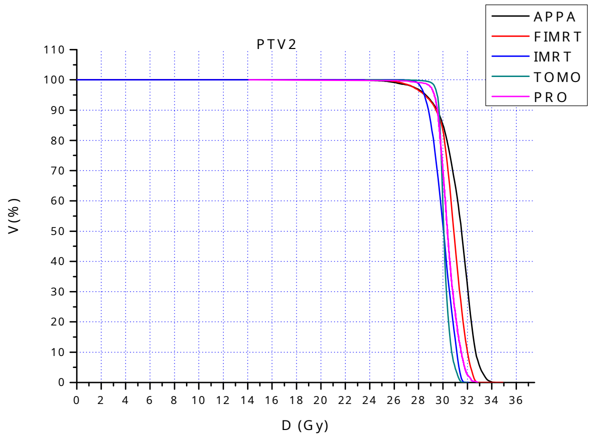

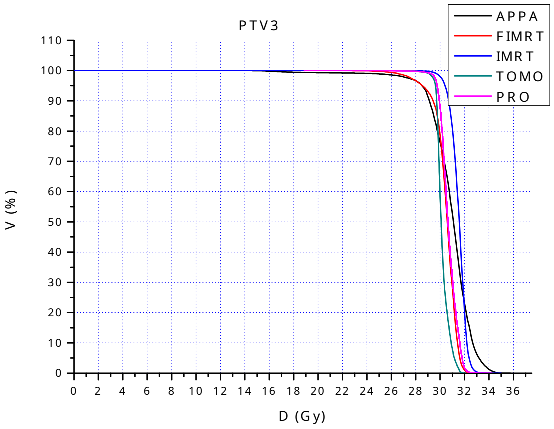

Supplement: Supplementary file 2 [file 1748-717X-8-22-S1.docx]
